# Time for a Paradigm Shift in Animal Nutrition Metabolic Pathway: Dietary Inclusion of Organic Acids on the Production Parameters, Nutrient Digestibility, and Meat Quality Traits of Swine and Broilers

**DOI:** 10.3390/life11060476

**Published:** 2021-05-24

**Authors:** Dhanushka Rathnayake, Hong Seok Mun, Muhammad Ammar Dilawar, Kwang Soo Baek, Chul Ju Yang

**Affiliations:** 1 Animal Nutrition and Feed Science Laboratory, Department of Animal Science and Technology, Sunchon National University, Suncheon 57922, Korea; dhanus871@gmail.com (D.R.); mhs88828@nate.com (H.S.M.); ammar_dilawar@yahoo.com (M.A.D.); 2 Interdisciplinary Program in IT-Bio Convergence System (BK21 PLUS), Sunchon National University, Suncheon 57922, Korea; gwangsu1649@daum.net

**Keywords:** organic acids, feeding, swine, broilers, digestibility, meat quality

## Abstract

Because the application of antibiotic growth promoters (AGP) causes accelerated adverse effects on the animal diet, the scientific community has taken progressive steps to enhance sustainable animal productivity without using AGP in animal nutrition. Organic acids (OAs) are non-antibiotic feed additives and a promising feeding strategy in the swine and broiler industry. Mechanistically, OAs improve productivity through multiple and diverse pathways in: (a) reduction of pathogenic bacteria in the gastro-intestinal tract (GIT) by reducing the gut pH; (b) boosting the digestibility of nutrients by facilitating digestive enzyme secretion and increasing feed retention time in the gut system; and (c) having a positive impact and preventing meat quality deterioration without leaving any chemical residues. Recent studies have reported the effectiveness of using encapsulated OAs and synergistic mechanisms of OAs combinations in swine and broiler productivity. On the other hand, the synergistic mechanisms of OAs and the optimal combination of OAs in the animal diet are not completely understood, and further intensive scientific explorations are needed. Moreover, the ultimate production parameters are not similar owing to the type of OAs, concentration level, growth phase, health status of animals, hygienic standards, and environmental factors. Thus, those factors need to be considered before implementing OAs in feeding practices. In conclusion, the current review evaluates the basics of OAs, mode of action, novel strategies to enhance utilization, influence on growth performances, nutrient digestibility, and meat quality traits of swine and broilers and their potential concerns regarding utilization.

## 1. Introduction

The ultimate goal in the global livestock sector is to achieve enhanced quantitative and qualitative productive parameters. A few decades ago, enhanced production was gained by incorporating various antibiotic growth promoters (AGP), which resulted in improved feed efficacy, growth rate, and lower mortality and disease. On the other hand, the emergence of antimicrobial-resistant bacteria has led to a discussion regarding the global health problem. Consequently, the utilization of AGP was banned by the European Union. Thus, scientists and researchers have focused on sustainable potential antibiotic-free production systems in the poultry sector [1] and swine industry [2].

**Figure 1:**
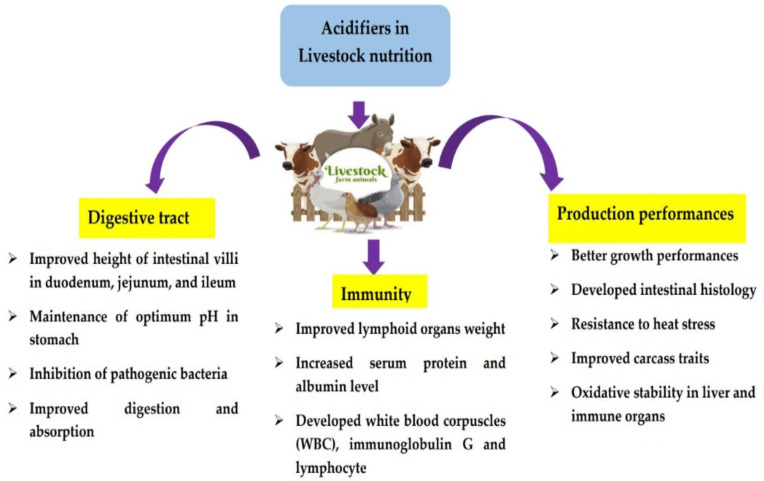
Various application and benefits of organic acids in the livestock sector [16].

Researches have highlighted the effective utilization of organic acids (OAs), phytobiotics, probiotics, prebiotics, bacteriophages, and other numerous alternatives instead of antibiotics to establish appropriate health and production parameters of animals. As a group of chemicals, organic acids can be defined as carboxylic acids including fatty acids, which have the chemical structure of R-COOH with specific chemical characteristics. They can be categorized into three groups: (a) simple mono-carboxylic acids (acetic, formic, propionic, and butyric acids); (b) carboxylic acids containing hydroxyl group (malic, lactic, tartaric, and citric acids); and (c) carboxylic acids with double bonds (sorbic and fumaric acids) [3]. OAs produce effective responses owing to their antimicrobial properties, which can enhance the pH reduction rate in the GIT [4]. Consequently, the intestinal digestibility and mineral utilization were improved [5,6]. Acidifiers were incorporated into animal diets a few years earlier owing to the presence of preservatives and nutritional characteristics [7,8]. Despite controlling the desirable growth rate of molds, fungi, and bacteria in animal feed, several studies have reported the potential ability of improving nutrition digestion and retention, intestinal health, and ultimate growth development of non-ruminant animals, including feed sanitizing characteristics [9,10,11]. Effective production parameters and health-promoting evidence have been discovered for numerous OAs, such as citric, fumaric, and formic acids and their salts [12]. Enhanced meat quality characteristics and growth performances were observed in broilers fed a diet supplemented with OAs, including 30% lactic, 25.5% benzoic, 7% formic, 8% citric, and 6.5% acetic acid [13]. Partanen and Morz [7] reported that incorporating OAs into the pig diet modulates the beneficial gut microbiota and improves the growth performance. A reduced gastric pH and retarded enterotoxigenic *E. coli* proliferation in the gut system occurred due to the inclusion of lactic acid into the pig diet. Thus, developed gut health led to optimal feed intake and weight gain of the animal [14]. Furthermore, supplementation of OAs with feedstuff will increase the stimulation rate of the nutrient digestion process [15]. The application of OAs in the livestock sector has produced numerous benefits in both economic and quality product perspectives in the livestock sector (Figure 1).

Each organic acid has a distinguished range of pH, antimicrobial potential, pKa values, and membrane structure. Especially, a combination of OAs has various pKa values directly influencing the intestine pH due to the developed synergistic effect [17]. The most common OAs involved in animal nutrition are listed below (Table 1).

This review evaluates the response of swine and broilers to OAs supplementation of previous studies in terms of the growth production parameters, including feed intake, weight gain, feed conversion ratio (FCR), nutrient digestibility, and meat quality traits. The possible modes of action, causes of various responses due to OAs, and potential concerns regarding OAs are also assessed.

## 2. Potential Modes of Action of OAs

OAs have numerous benefits on the health and development of the gut system. Nevertheless, the mode of action is not completely understood. Their modes of action may be attributed partially to different factors, such as (A) mineral chelation and stimulation on intermediary metabolism; (B) inhibition of the development of pathogenic microbes; (C) facilitation of proper digestion due to lower gastric pH and enhanced pepsin secretion; and (D) reduction of gastric emptying rate and maintenance of endogenous enzymes secretion [4,19].

### 2.1. Effect of OAs on Mineral Utilization and Nutrient Digestibility

OA anions form complexes with Mg, P, Ca, and Zn, improving digestion and minimizing the excretion of beneficial minerals from the body. Phytate phosphorous utilization occurs through OAs administration by providing favorable pH conditions to convert phytase into hydrolyze phytate [20]. Bolling et al. [21] reported that citric acid facilitates the removal of attached minerals to phytate molecules, such as Ca, P, and Zn. Furthermore, it has been found that fumaric acid also can enhance the apparent absorption and retention of Ca, P, and Zn in the gastro-intestinal tract (GIT) [22].

The inclusion of OAs into the diet can improve energy utilization of certain feedstuffs. In soybean meal, for instance, the lower metabolizable energy (ME) in soybean meal occurs due to retarded digestibility in the carbohydrate portion. However, the presence of endogenous α-(1,6)-galactosidase enzyme in the intestines will facilitate the proper digestion of carbohydrate portion in the soybean meal. Ao [23] reported that the inclusion of 2% citric acid increased the digestion process by enhancing the activity of the α-galactosidase enzyme. Moreover, by reducing the chime pH, OAs supplementation improved protein digestion owing to microbial phytase activity and induced pepsin secretion [24]. A further protein digestion process improves through the secretion of a greater level of chymotrypsinogen A, chymotrypsinogen B, procarboxy peptidase A, procarboxy peptidase B, and trypsinogen enzymes [25]. OAs inclusion also increased the proper absorption rate of nutrients in the GIT by increasing the digesta retention time [26]. The increased ME and crude protein (CP) were observed due to the reduced microbial competition with the host, ammonia emission, and endogenous nitrogen elimination [27].

### 2.2. Effect of OAs on Antimicrobial Activity and Pathogenic Bacteria

**Figure 2:**
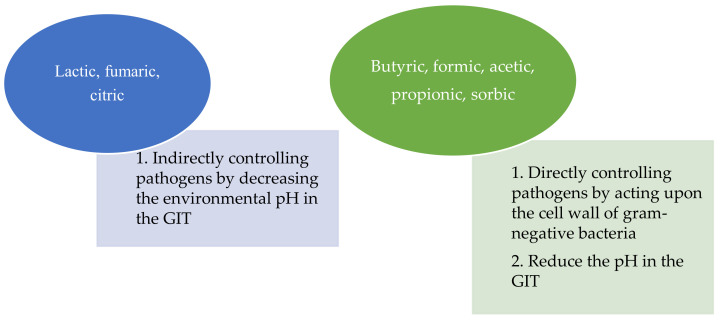
Two different mechanisms of organic acids on altering the pH of the GIT and their impact on pathogens [15].

Both animals and plants have symbiotic relationships with various microbes to survive in the environment through an active defense system against pathogens and to regulate the metabolism associated with hormones. On the other hand, an excessive microflora content produces unnecessary competition between the host and nutrition. Hence, maintenance of the optimal microbe composition in the GIT should be investigated. Some OAs can alter the GIT by eradicating foodborne pathogens, such as *Salmonella* and *E. coli* species [28]. Owing to pH reduction and their influence on the buffering capacity of the diet, OAs can improve gut health by providing the optimal environment to beneficial microbes while preventing the proliferation of pathogens [7,29,30]. OAs can be divided into two groups based on the microbial ameliorate capacity in the GIT (Figure 2).

Non-disassociated OAs enter the cytoplasm through the semipermeable membrane of the microorganism. Thereafter, OAs release their protons (H^+^), and the cytoplasm pH decreases gradually. The enzymes involving reactions, such as nutrient transportation and glycolysis signal transductions of the microbes, are curtailed. Consequently, an energy deficiency occurs to maintain the normal pH [31]. Owing to the acidic conditions in the stomach, the efficacy of OAs is greater than under neutral pH conditions, as in the intestines. On the other hand, most bacteria species require optimal environmental pH conditions and pH < 4.5 (extreme lower) conditions, which adversely affect their survival. By releasing H^+^ ions, OAs aid in the dysfunctions, retardation, or inhibition of the multiplication of pH-sensitive bacteria [32].

Some bacteria in the GIT secrete various harmful compounds that reduce fat digestibility, stimulate rapid turnover of absorptive epithelial cells, stimulate mucus secretion, and induce an inflammatory responses due to the developed immune system. These factors help retard the growth performance, and approximately 6% net energy losses in pigs can be attributed to microflora [33]. OAs have the potential to eliminate specific species, such as *Coliforms*, while generating eubiosis. Thus, they can provide the optimal microbial atmosphere in the GIT that can benefit the host by accumulating lower toxic compounds, amines, and ammonia. While the gram-positive (G^+^) bacteria are susceptible to long-chained OAs, the gram-negative (G^−^) bacteria cannot resist OAs with fewer than eight carbons [34]. However, stronger effects of OAs affect G^+^ bacteria due to cell structural differences. In contrast, the cytoplasmic membrane of bacteria is surrounded by a thick peptidoglycan layer. Generally, this peptidoglycan layer is thicker in G^+^ bacteria than G^−^ bacteria. Nevertheless, G^−^ bacteria have an extra lipopolysaccharide layer that is more resistant to hydrophobic antibiotics and chemical compounds.

**Figure 3:**
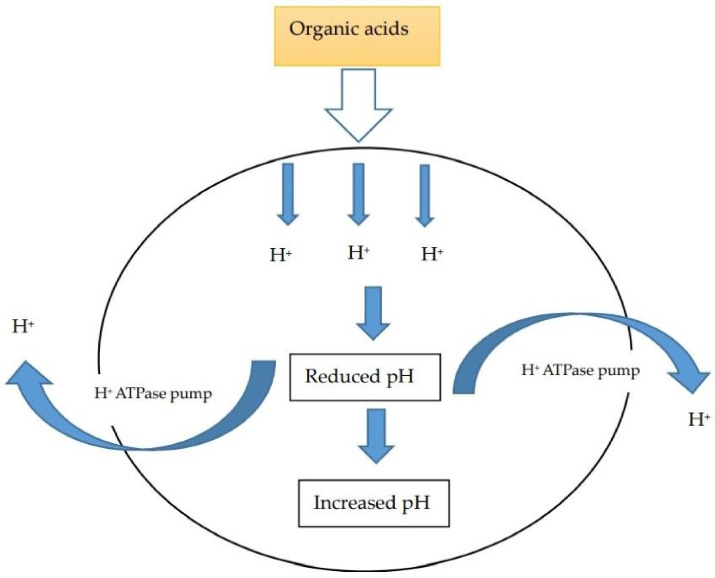
Mode of action of organic acids on pH-sensitive bacteria (*Clostridia*, *Salmonella*, *Coliforms*, *Listeria* spp.) [36].

Because OAs have both bactericidal and bacteriostatic properties, Luckstadt and Mellor [35] sketched out the mode of action of OAs on G^−^ bacteria as follows: (1) Lipophilic undissociated OAs penetrate the G^−^ bacteria cytoplasm (*Salmonella*). (2) OAs release H^+^ ions, which reduces the cellular pH, and the enzyme-based microbial metabolism tends to decrease (3). To restore the normal cytoplasmic pH, the cell is forced to discard H^+^ ions through the cell membrane via the H^+^- ATPase pump. (4) Ultimately, G^−^ bacteria proliferation is gradually impeded when exposed to OAs for some time (Figure 3).

This anion model of OAs can vary upon two factors: (1) the lipophilic nature of the OAs, which can transmit through the microbes cell wall; and (2) various anion complexes involved with different inhibitory actions within the cell [37,38].

## 3. Novel Strategies for Enhancing Efficacy of OAs Availability in the Gastrointestinal Tract (GIT)

The supplementation of feed and water with OAs did not show better efficacy in the latter part of the GIT. It occurs due to the high proportion of short-chain fatty acids (SCFA) that are metabolized and absorbed rapidly in the upper segments of the GIT [39]. Thus, the OAs concentration tends to decrease and exert negative feedback on modifying the host microflora content in the GIT. New studies have shown that microencapsulated lipid shells facilitate the transportation of SCFA further down the GIT while increasing the retention time [39,40]. Furthermore, microencapsulated feed improves palatability, removes unpleasant odors/ flavors, and delivers the necessary compounds to the specific target place inside the animal body while acting as a “biological agent” [2]. Further studies have demonstrated that the efficiency of OAs can be improved by incorporating phytogenic feed additives. Owing to the synergistic effects of both OAs and botanicals, food-safety bacteria, such as *C. jejuni* and *S. typhimurium* counts, tend to decrease. In particular, pore-forming agents derived from numerous aromatic compounds caused changes in the bacterial cell membrane by providing a pathway for OAs entrance [41,42]. Grilli et al. [43] indicated that a combination of microencapsulated OAs (citric and sorbic acid) and pure botanicals in weaning pigs’ diet improved the maturation of the intestinal mucosa by exerting a positive impact on the barrier integrity in the jejunum and ileum while performing better growth performance. Gheisar et al. [44] observed a higher *Lactobacilli* content in the feces of broilers fed a diet supplemented with 0.075% microencapsulated OAs. Gheisari et al. [45] also reported that 0.2% OAs inclusion enhanced the *Lactobacilli* content while reducing the *Clostridium perfringens*, *E. coli*, and *Salmonella* spp content. Moreover, a 0.5% microencapsulated blend of OAs reduced the oxidative status, microbial loads, and improved the shelf life of broiler meat [46]. The protected OAs could be delivered to specific sites in the body and had a positive effect by eliminating the *Coliforms* counts in both the distal jejunum and cecum. In contrast, freely available OAs had little influence [47].

Since the form of OAs (SCFA) is naturally produced by the GIT, some studies reported that introducing both prebiotics and probiotics could stimulate the synthesis of SCFAs in the GIT [48,49]. This could be implemented in two ways: (1) direct administration of lactic acid-producing bacteria in the diet; and (2) the addition of prebiotic substances that enhance the proliferation of lactic acid bacteria and increase SCFA production [50,51]. *Streptococcus*, *Saccharomyces*, *Enterococcus*, *Lactobacilli*, *Bacillus*, *Bifidobacterium*, and *Pediococcus* are the probiotic strains mainly used in nutritional studies. These microbes protect the GIT through barrier effects, competitive exclusion, bacterial interference process, prevention of colonization, and bacterial antagonism [52,53]. These probiotic supplementations develop the cell structure, have immunological effects, and are resistant to pathogens. Ultimately, they enhance the production rate of SCFAs, intermediary products, and H_2_O_2_. *Lactobacilli* spp. can generate lactic acid, which can facilitate the synthesis of butyric acids with the collaboration of *Clostridial* clusters that reinforce the animal cross-feeding process [48]. Oligosaccharides and polysaccharides (non-digestible carbohydrates (NDC)), proteins, and particular lipids are used as prebiotics to provide a proper substrate for beneficial host microbes. Previous studies have stated that the administration of prebiotics has a positive effect on the natural production of OAs in the GIT. The supplementation of the diet with NDC enhances the *Lactobacilli* and *Bifidobacterium* population. In contrast, these microbes promote the synthesis of SCFA, such as propionate, acetate, and butyrate acids [54]. Rehman et al. [55] reported that 1% inulin inclusion increased the jejunum acetate concentration and n-valerate in the cecal digesta in broilers compared to the control diet. Overall, the dietary supplementation of prebiotics and probiotics will lead to optimal OAs production in the animal body.

## 4. Effect of OAs in Swine and Broiler

### 4.1. Supplementation of OAs on the Growth Performance of Swine and Broilers

Studies have found that the optimal dosage of OAs can enhance the productivity of pigs compared to AGPs. Increased growth performance, gain to feed (G: F), and feed intake (FI) was observed in piglets supplemented with an OAs mixture (benzoic, fumaric, lactic, propionic, and citric) [56]. Because benzoic acid in the diet can increase the butyric acid concentration in the GIT, the gut microflora ameliorating process occurs by acting as an energy source agent in gut epithelial cells [57]. The feed conversion ratio (FCR) was increased by 10%, and the average daily gain (ADG) was increased by 3% when pigs were administered fumaric and citric acids at four weeks of age [58]. Kuang et al. [59] reported that the inclusion of an OAs blend (calcium formate, calcium lactate, and citric acid) and medium-chain fatty acids (MCFAs—lauric, capric, and myristic) enhanced the FI, ADG, and FCR in weaning pigs compared to pigs fed with dietary zinc oxide inclusion. Feeding weaning pigs with 0.8% fumaric acids reduced the *E. coli* and *Coliforms* population in the cecum [60]. On the other hand, Risley et al. [61] reported that 1.5 % fumaric acid addition did not affect the microflora composition in the GIT. According to the study conducted by Htto et al. [62], the ADG, FCR, and body weight (BW) during 0–14 days (piglets) were not significantly different from those fed OAs. In the same study, however, the ADG and FCR were enhanced during 15–35 days and, overall, 35 days of periods due to salts of organic acid (potassium diformate and potassium formate) supplementation. Furthermore, a trend of developed growth performance in response to the inclusion of OAs combined with salts combination was more reliable in growing-finishing pigs than in weaning pigs [6]. Canibe et al. [63] and Partanen et al. [64] reported increased ADG and G: F ratios in pigs fed a diet containing formic acid, ammonium formate, and formic acids. A combination of phytogenic feed additives with organic acids (10% citric, 10% sorbic, 6.5% malic, and 13.5% fumaric acid) also improved the BW and ADG of weaning pigs. Moreover, Yang et al. [65] reported that a high abundance of *Limosilactobacillus mucosae* also occurred compared to the control treatment. Nevertheless, 1.8% formic acid inclusion did not have a positive response on the ADG and average daily feed intake (ADFI) of weaning pigs, but it enhanced the G: F [63]. The above dissimilarities among the different studies might be related to the inclusion dosage of OAs, diet complexity, growth phase, and animals’ health conditions (Table 2). Therefore, further studies will be needed to identify the best OAs concentration for different growth stages.

In the broiler growth performance, the utilization of OAs has not gained as much attention as in the swine industry. The rapid metabolization process in crops to the gizzard (foregut) causes a deficiency of OAs availability and retards the growth performance [33]. However, Fascina et al. [13] reported that the administration of OAs combination (30% lactic, 25.5% benzoic, 7% formic, 8% citric, and 6.5% acetic acid) improved the BW, weight gain (WG), and FCR compared to the control group at 42-day-old broilers. Broilers fed OAs-supplemented diets showed a significant (*p* < 0.05) improvement in the FCR due to better utilization of nutrients [66]. Hassan et al. [67] reported that microencapsulated galliacid OAs mixture (fumaric acid, calcium formate, calcium propionate, potassium sorbate, and hydrogenated vegetable oil) enhanced the WG by 16% compared to the control groups. Kamal and Ragaa [68] also indicated that in 42 days old broilers, the BWG and FCR were enhanced in those fed 3% organic acids (butyric, fumaric, and lactic acid). Hence, the higher BWG was achieved through direct antimicrobial effect, reducing the digesta pH level in the GIT while acting as a barrier to pathogens and buffering reactivity in conjunction with the enhanced nutrient digestibility [69]. Interestingly, the synergistic effects of combined 0.3 g/kg essential oils (thymol, vanillin, and eugenol) with encapsulated OAs (fumaric, sorbic, malic, and citric) increased the FCR significantly while minimizing the *E. coli* population through a lower gut pH value [70].

**Table 2:** Effect of organic acids mixture on growth performances and other parameters of swine and broilers.

Dosage and Organic Acid/Acids	Growth Phase	Growth Performances			Intestinal/Fecal Microbial Counts (CFU)	Other Parameters	References
		BWG/FBW	ADFI	G:F			
**Swine**							
0.1% and 0.2% fumaric, citric, malic, MCFA (capric and caprylic)	Weaning	S	NS	S	*E. coli*; S *Lactobacilli*; S *Clostridium*; S *Salmonella*; S	Reduced diarrhea score, fecal ammonia, and acetic acid emission	Yang et al., 2018 [71]
0.1% and 0.2% fumaric, citric, malic, MCFA (capric and acrylic)	Growing	S	NS	S	*Lactobacilli*; S *E. coli*; NS	-	Upadhya et al., 2016 [72]
0.15% benzoic, fumaric, calcium formate	Weaning	S	NS	NS	*E. coli*; NS *Lactobacilli*; NS	Increased villus height in duodenum and jejunum Increased butyric acid level in the cecum and valeric acid level in the colon	Xu et al., 2017 [73]
1.1% acetic, propionic, phosphoric, citric acid	Weaning	NS	NS	NS	*Lactobacilli*; NS *E. coli*; NS *Coliforms*; NS	Reduced pH level in colon Retardation of *Coliforms* proliferation	Namkung et al., 2004 [74]
0.4% and 0.2% fumaric, lactate, citric, propionic, benzoic acid	Weaning	NS	NS	NS	*E. coli*; NS	-	Walsh et al., 2007 [56]
0.5% benzoic acid	Weaning	S	S	S	*Lactobacilli*; S	-	Wei et al., 2021 [75]
0.5, 1% benzoic acid	Weaning	S	NS	NS	NE	Reduced the number of aerobic, total anaerobic, lactic acid-forming, and gram-negative bacteria in the stomach Reduced gram-negative bacteria and acetic acid in the duodenum Reduced gram-negative bacteria in ileum	Kluge et al., 2005 [76]
0.5% butanoic, fumaric, benzoic acid	Piglets	S	NS	S	*Lactobacilli*; NS *E. coli*; NS	Decreased ileal *E. coli* bacteria level Did not exert negative impacts on GIT pH level and immunity	Li et al., 2008 [77]
0.1% fumaric, citric, malic, MCFA (capric and caprylic)	Finishing	S	NS	S	*Lactobacilli*; NS *E. coli*; NS	Reduced feces H_2_S gas emission	Upadhya et al., 2014 [78]
0.85% formic, benzoic, sorbic, Ca- butyrate	Growing male pigs	NS	NS	NS	*E. coli*; S *Lactobacilli*; S	Lower level of *Coliforms*, *Enterococci*, and lactic acid bacteria in jejunum and colon descendens	Øverland et al., 2007 [79]
0.5% benzoic acid	Weaning	S	S	S	*E. coli*; NS *Lactobacilli*; NS	Reduced diarrhea in weaning pigs	Papatsiros et al., 2011 [80]
0.14% and 0.64% formic acid	Weaning	S	S	NS	*Lactobacilli*; S	Higher microbiota diversity in 0.64% dosage	Luise et al., 2017 [9]
**Broilers**							
0.3% and 0.4% calcium formate, calcium propionate 0.3, 0.4% ammonium formate, ammonium propionate	Finishing	S	NS	S	NE	Reduced the ileal total bacterial count Improved villi length	Saleem et al., 2020 [81]
1% formic, lactic, propionic, citric acid	Finishing	S	NS	NS	NE	Enhanced V: C in GIT Increased water consumption during 15–22 days	Ali et al., 2020 [82]
0.5% citric, sorbic, synthetic essential oil	Finishing	NS	NS	NS	*E. coli*; NS *Enterococci*; S *Clostridium*; NS *Enterobacteriaceae*; NS	Increased villi height, crypt depth, number of villi, mucosa thickness, and villi area	Stamilla et al., 2020 [83]
0.15% formic, lactic, citric, malic, tartaric, phosphoric acids	Finishing	S	S	S	*Lactobacilli*; S *E. coli*; S	Enhanced inhibitory action owing to organic acid	Goh et al., 2020 [84]
0.3% formic, acetic, propionic, ammonium formate	Finishing	S	NS	NS	NE	Increased SCFAs level in the cecum Increased jejunal goblet cell density and ileal villus height	Dai et al., 2021 [85]
0.1% lactic, citric, acetic, formic, propionic, phosphoric, and sodium butyrate	Finishing	S	NS	S	*Lactobacilli*; S *Coliforms*; NS	Increased jejunum villus height Enhanced humoral immune response	Sabour et al., 2018 [86]
0.3, 0.5% formic, propionic acid	Finishing	S	NS	S	*Lactobacilli*; S *E. coli*; S	Lower duodenal pH High immune response against Newcastle disease, infectious bronchitis	Fathi et al., 2016 [87]
0.06% fumaric, calcium format, calcium propionate, potassium sorbate, hydrogenated vegetable oil	Finishing	S	S	S	*Lactobacilli*; S *Salmonella*; S	Increased dressing percentage and bursa weight	Hassan et al., 2010 [67]
0.2, 0.4, and 0.6% butyric acid	Finishing	S	NS	S	*E. coli*; S	pH reduction of upper GIT Increased villus length and crypt depth in the duodenum	Panda et al., 2009 [88]
0.5, 1, 1.5, and 2% citric, lactic, phosphoric acid	Finishing	S	NS	S	*E. coli*; S *Salmonella*; S	2% OAs blend enhanced the carcass yield 1.5%, 2% OAs blend increased the liver weight	Sultan et al., 2015 [89]
0.6% formic acid	Finishing	S	NS	S	*E. coli*; S (in crop)	Higher digestibility of crude protein, high dressed yield, and lower fat content in carcass	Panda et al., 2009 [90]
2% butyric, fumaric, lactic, and 3% butyric, fumaric, lactic acid	Finishing	S	NS	S	NE	Increased villus height in the small intestines Enhanced serum calcium and phosphorus concentrations	Adil et al., 2010 [25]
0.2% propionic, 0.3% butyric acid	Finishing	S	NE	S	NE	Increased tibia weight, tibia length	Lakshmi and Sunder., 2015 [91]

BWG, body weight gain; FBW, final body weight; ADFI, average daily feed intake; G: F, gain to feed ratio; S, significant; NS, non-significant; NE, not evaluated.

### 4.2. Supplementation of OAs on Nutrient Digestibility of Swine and Broilers

Generally, OAs have been utilized as an acidifier in the livestock feeding sector. Furthermore, OAs inclusion is considered to be an effective antibiotic alternative for enhancing nutrient digestibility. Microencapsulated OAs, including 10% malic, 13% citric, and 17% fumaric acids, enhanced the digestion of nitrogen (N), dry matter (DM), and energy in finishing pigs and lactating sows [78,92,93] and significantly increased DM, crude protein (CP), fat, and energy digestibility in growing pigs [94]. The supplementation of 1.5% citric acid increased the coefficient of the total tract digestibility (CTTAD) of crude protein (CP), calcium (Ca), and phosphorous (P) of sows during the late gestation and lactation period [95]. Moreover, Yang et al. [71] stated that protected OAs incorporation has positive effects on the apparent total tract digestibility (ATTD) of (DM) in weaning pigs. The inclusion of benzoic acid resulted in improved apparent digestibility of Ca and P in growing pigs [96,97]; CP digestibility in weaning pigs [98]; and the DM, CP, ether extract (EE), and crude fiber (CF) of sows [99]. Previous studies demonstrated positive influence of OAs supplementation on the nutrient digestibility in both swine and broilers (Table 3).

Nevertheless, Upadhya et al. [72] found no significant differences in DM, N, and energy digestibility owing to dietary supplementation of OAs in three different concentration levels.

Since OAs were used as an alternative feed additive, they helped improve the growth performance and productivity parameters in broilers. The addition of 0.5% and 1% formic acid into the finisher diet enhanced apparent ileal digestibility (AID) of DM and CP compared to the control treatment [100,101]. Ao et al. [102] reported that 2% citric acid incorporation improved the retention of DM, CP, and neutral detergent fiber content in the GIT. In one study, the gross energy, CP, and EE digestibility of broilers at nine days of age was found to be 78.01, 76.07, and 72.85% in the 200ppm ascorbic acid-supplemented group, which was significantly higher as compared with 76.20, 72.62, and 67.65% in the non-supplemented group, respectively [103]. The supplementation of 0.2% OAs with a phytase combination more significantly enhanced the CP (88.58%) and EE (85.61) digestibility in chicks than the CP (77.51) and EE (79.49) values in the control treatment [104].

Smulikowska et al. [105] reported that fat-coated OAs inclusion improved the N retention, organic matter (OM), and metabolizable energy corrected for nitrogen (AMEn) values. The reason for the higher N retention might have been the improved epithelial cell proliferation in the GIT, while non-protected OAs tended to be metabolized rapidly [106]. Owing to the synergistic effect, combined OAs and essential oil (EO) administration into the broiler diet enhanced the AID of DM and energy at 21 days of rearing [107]. Nevertheless, the expected synergism effect was not observed from the combination of citric acid with microbial phytase. The non-significant impact on the AID of CP and amino acids (AA) might be associated with the formation of complexes among citric acids with Ca and the subsequent decrease in binding ability with phytate allowing easy hydrolyzation by the enzymes.

**Table 3:** Effect of organic acids combination on nutrient digestibility of swine and broilers.

Dosage and Organic Acid/Acids	Growth Phase	Digestibility				Reference
		DM	N	E	CP	
**Swine**						
0.2% fumaric, citric, malic, capric, and caprylic acid	Growing	S	S	S	S	Hossain et al., 2011 [94]
0.05% citric, sorbic acid	Growing	S	NS	S	NC	Cho et al., 2014 [108]
2% benzoic acid	Lactating sows	S (OM)	NE	NE	S	Kluge et al., 2010 [99]
0.1% and 0.2% fumaric, citric, MCFA	Finishing	S	S	S	NE	Upadhaya et al., 2014 [92]
0.5% phenyllactic acid	Weaning	S	S	NE	NE	Wang et al., 2009 [109]
0.3% formic, acetic, propionic, MCFA	Weaning	S (DM) NS (OM)	NS	NS	NS	Long et al., 2018 [110]
0.5% formic, propionic, lactic, citric, sorbic acid	Post-weaning	NS	NS	NS	NS	Gerritsen et al., 2010 [111]
300 mEq acid/kg formic, n-butyric acid	Growing	S	S	S	S	Mroz et al., 2000 [112]
0.15% citric acid	Lactating sows	NE	NE	NE	S	Liu et al., 2014a [95]
0.2% fumaric, citric, malic, capric, caprylic acid	Lactating sows	S	S	S	NE	Devi et al., 2016 [113]
** Broilers **						
0.2% formic, propionic acid	Finishing	NS	NE	NE	S	Emami et al., 2013 [104]
0.5% formic acid	Finishing	NS	NE	NE	NS	Hernández et al., 2006 [101]
0.25, 0.5, and 0.75% formic acid	Finishing	NS	NE	NE	S	Ndelekwute et al., 2015 [114]
5000ppm and 10,000ppm formic acid	Finishing	S	NE	NE	S	Garcia et al., 2007 [101]
0.25% acetic, butyric, citric, formic acid	Finishing	S	NE	NS	S	Ndelekwute et al., 2019 [115]
1, 2, and 3% citric acid	Finishing	NE	NE	S	S	Ghazalah et al., 2011 [69]
0.5, 1, and 1.5% fumaric acid	Finishing	NE	NE	S	S	Ghazalah et al., 2011 [69]
0.25, 0.5% formic acid	Finishing	NE	NE	NS	S	Ghazalah et al., 2011 [69]
0.25, 0.5, and 0.75% acetic acid	Finishing	NE	NE	S	NS	Ghazalah et al., 2011 [69]

N, nitrogen; E, energy; CP, crude protein; S, significant; NS, non-significant; NE, not-evaluated; OM, organic matter; DM, dry matter.

### 4.3. Effect of OAs Supplementation on Meat Quality on Pigs and Broilers

Few studies have investigated the meat quality parameters based on the incorporation of OAs in animal diets. Moreover, an examination of the meat quality traits of pigs and broilers is important because the consumption of quality meat has gained an important place in the food industry. Upadhya et al. [92] reported that supplementation of an OAs blend (consisting of fumaric, citric, malic, and MCFA) did not have adverse effects or improvements in the meat color, pH, cooking loss, drip loss, and water holding capacity (WHC). Similarly, Cho et al. [108] reported that the administration of a microencapsulated OAs combination, including citric and sorbic acids, did not significantly affect the meat color, pH, sensory attributes (color, firmness, marbling), cooking loss, and WHC. In contrast, the inclusion of 0.05 and 0.1% fumaric, citric, malic, and MCFAs resulted in lower drip loss in pork (22.05%) except for any differences in meat color, sensory evaluation, cooking loss, pH, and WHC [116]. Jansons et al. [117] reported a higher protein content (21.94%) in *longissimus lumborum* muscle tissues and lower cholesterol content (51.1 mg/kg^−1^) in pork after the addition of formic, acetic, citric, phosphoric acid along with phytogenic feed additives to the diet. This might be attributed to the synergistic effect and the presence of antioxidant compounds in the feed. However, further investigations will be needed to determine the possible mode of actions associated with the meat quality characteristics by introducing OAs to the animal diet.

Brzóska et al. [118] reported that the supplementation of OAs to a broiler diet resulted in no significant influence on breast muscle content and leg muscle weight. The chemical constitutes of the leg meat, including DM, protein, and fat content, also did not vary due to OAs application. Nevertheless, Jha et al. [119] reported that the inclusion of OAs (formic + propionic acid, formic + citric acid, formic + sorbic, and formic+ lactic acid) enhanced the meat thigh weight (29.03%), back weight (53.4%), wings weight (31.27%), and breast weight (34.57%) compared to the control group. On the other hand, they did not evaluate any other meat quality parameters regarding OAs inclusion. Supplementation at the recommended dosage of an acetic, butyric, formic, phosphoric, lactic acid blend did not have significantly favorable results on carcass pH, shear force, WHC, cooking loss, and meat color values, but the TBARS value was increased significantly in birds fed with an OAs mixed diet (2.01 nmol MDA/mg) as compared with control group (1.10 nomol MDA/mg). This suggests that a higher fat content facilitated a higher lipid oxidation process in meat [120]. Meat pH has a significant influence on WHC, meat color, juiciness, tenderness, and shelf-life. The changes of meat pH result from post-mortem metabolism and the conversion of glycogen into lactic acid [121]. In contrast, at a lower pH range (pH < 5.8), broiler meat exhibited a pale, soft, and exudative (PSE) condition, which is considered a degraded meat quality parameter compared to meat exposed to higher pH levels (pH > 5.8) [122]. Sugiharto et al. [123] found that a higher meat pH (6.7%) in broilers occurred in a diet administered with 0.1% formic and 0.3% butyric acid compared to the control group. El-Senousey et al. [124] presented a possible reason for the OAs and higher meat pH occurrence: the decline in post-mortem muscle glycolysis inhibited the decrease in muscle pH after slaughter. Furthermore, lower drip loss and a lightness value were reported in the diet combined with both formic and butyric acid but decreased due to the single administration of butyric acid. This might be due to the distinctive characteristics of each OA and the metabolic activities of each associated with specific pKa. Menconi et al. [125] reported less drip loss (65.85%) in broiler meat with feeding blends of lactic, tannic, caprylic, propionic, acetic acids, and butyric acid. Nevertheless, inconsistent results were obtained by Attia et al. [126], who reported a decrease in WHC (26.45%) in broiler meat owing to the supplementation of citric and fumaric acids. These results were attributed to differences in the OAs type, dosage, and experimental environment.

Nutritional quality of meat can also be influenced by the feeding strategies. Akbar et al. [127] observed a significantly higher PUFA content and lower SFA proportion of the birds fed a diet supplemented with organic acid salts (1% calcium propionate), which is beneficial, from the human nutrition point of view, as lower saturated fatty acid (SFA) and higher (PUFA) may positively influence human health. Furthermore, a lower cholesterol content was also reported in the diet containing dietary OAs.

## 5. Potential Concerns Regarding OAs Utilization

Pathogenic microbial activities encompass a broad range of survival mechanisms that facilitate the multiplication and survival by evading their host defense system. Although OAs supplementation has a favorable impact on pigs and broiler production through the biological defense system, bacterial resistance should be considered. Recent studies revealed the specific, developed mechanisms of *Escherichia coli* [128] and *Salmonella enterica* [129,130]. Mutations of some genes, such as wecA (rfe), waaG (rfaG), fcl (Fucose, FX-like), and wecB (rffE), influence creating adaptation ability in *Salmonella* spp and *E. coli* groups against the OAs. This adaption process consists of two mechanisms: (1) transient adaptation and (2) pre-challenge adaptation, which occurs specifically in lower pH ranges [131,132,133]. On the other hand, the prevalence is affected by the temperature and type of the acidifier. OAs, being SCFA, have lower pH levels in the GIT, consequently reducing the livability of pathogenic bacteria at the cellular level. Nevertheless, bacterial pH-independent tolerance is also possible. Higher resistance to *S. typhimurium* was observed when the animals were exposed to higher SCFA concentrations, which produced a lower pH environment in the GIT [134]. Another considerable effect of supplementation of OAs is the reduction of lactic acid synthesis and lactic acid bacteria population in the GIT. Thompsan and Hinton [135] reported the interaction of formic and propionic acid inclusion on lower lactic acid production in the crop. Thus, the concentrations and interactions among each OA combination may require further molecular-based studies to identify the associated potential risks.

## 6. Conclusions

The supplementation of organic acids has significant effects on the growth performance and digestion of nutrients by modulating the gut environment of both swine and broilers. According to the literature, OAs supplementation effectively induces a pH reduction in the GIT, plays a significant role against pathological bacteria colonization, and eventually improves nutrients utilization and growth performances. Incorporation of microencapsulation technology has potential ability to enhance the uniform transportation of SCFA further down the GIT while allowing effective utilization. Moreover, prebiotics and probiotics inclusion also influence enhanced SCFA availability through beneficial gut microflora activities. Further, acting as an energy source during GIT intermediary metabolism, OAs inclusion positively influences growth performances. The growth performances and carcass quality development occur due to proper nutrient utilization, feed conversion efficiency, and improved weight gain. On the other hand, to benefit from the effectiveness of OAs, the inclusion dosage, rearing environment, nutrient composition, growth phase, and health status of animals need to be considered in future studies. The chemical mechanisms of OAs in animal diet and synergism effects are not completely understood. Therefore, it is impossible to recommend a specific combination of OAs and concentrations that will positively affect the swine and broiler production parameters and meat quality traits. Once more knowledge becomes available, promising feeding strategies can be implemented by incorporating various organic acid formulas into the animal diet. In a nutshell, OAs supplementation to the animal diet exerts a positive impact on growth performances, inhibition of pathogenic bacteria, enhancement of nutrient digestibility, meat quality characteristics, and mineral utilization by minimizing microbial competition.

## Figures and Tables

**Table 1:** Common organic acids involved in animal nutrition and their properties [16,18].

Acid	Chemical Name	Registration Number	Molecular Weight/GE (MJ/Kg)	Odor	pKa
Butyric	Butanoic Acid	-	88.12/24.8	rancid	4.82
Citric	2-Hydroxy-1,2,3-Propanetricarboxylic Acid	E 330	192.1/10.2	odorless	3.13
Propionic	2-Propanoic Acid	1a297	74.08/20.6	pungent	4.88
Sorbic	2,4-Hexandienoic Acid	E 200	112.1/27.85	mildly acrid	4.76
Formic	Methanoic Acid	E 236	46.03/5.7	pungent	3.75
Acetic	Ethanoic Acid	E 260	60.05/14.6	pungent	4.76
Lactic	2-Hydroxypropanoic Acid	E 260	90.08/15.1	sour milk	3.83
Malic	Hydroxybutanedioic Acid	E 296	134.1/10.0	apple	3.40
Fumaric	2-Butenedioic Acid	2b08025	116.1/11.5	odorless	3.02
Benzoic	Benzenecarboxylic acid	-	-	-	4.20

## Data Availability

Not Applicable.

## References

[1] Nguyen D.H., Kim I.H. (2020). Protected Organic Acids Improved Growth Performance, Nutrient Digestibility, and Decreased Gas Emission in Broilers. Animals.

[2] Tugnoli B., Giovagnoni G., Piva A., Grilli E. (2020). From Acidifiers to Intestinal Health Enhancers: How Organic Acids Can Improve Growth Efficiency of Pigs. Animals.

[3] Shahidi S., Yahyavi M., Zare D.N. (2014). Influence of Dietary Organic Acids Supplementation on Reproductive Performance of Freshwater Angelfish (Pterophyllum Scalare). Global Vet..

[4] Kim Y.Y., Kil D.Y., Oh H.K., Han I.K. (2005). Acidifier as an Alternative Material to Antibiotics in Animal Feed. Asian Australas. J. Anim. Sci..

[5] Kil D.Y., Piao L.G., Long H.F., Lim J.S., Yun M.S., Kong C.S., Ju W.S., Lee H.B., Kim Y.Y. (2005). Effects of Organic or Inorganic Acid Supplementation on Growth Performance, Nutrient Digestibility and White Blood Cell Counts in Weanling Pigs. Asian Australas. J. Anim. Sci..

[6] Mroz Z., Reese D.E., Øverland M., Van Diepen J.T.M., Kogut J. (2002). The effects of potassium diformate and its molecular constituents on the apparent ileal and fecal digestibility and retention of nutrients in growing-finishing pigs. J. Anim. Sci..

[7] Partanen K.H., Mroz Z. (1999). Organic acids for performance enhancement in pig diets. Nutr. Res. Rev..

[8] Spratt C.D. (1985). Effect of Mould Inhibitor Treated High Moisture Corn on Performance of Poultry. Master’s Thesis.

[9] Luise D., Motta V., Salvarani C., Chiappelli M., Fusco L., Bertocchi M., Mazzoni M., Maiorano G., Costa L.N., Van Milgen J. (2017). Long-term administration of formic acid to weaners: Influence on intestinal microbiota, immunity parameters and growth performance. Anim. Feed. Sci. Technol..

[10] Ndelekwute E.K., Assam E.D., Ekere P.C., Ufot U.E. (2016). Effect of organic acid treated diets on growth, apparent nutrient digestibility and faecal moisture of broiler chickens. Niger. J. Anim. Prod..

[11] Huang C., Song P., Fan P., Hou C., Thacker P., Ma X. (2015). Dietary Sodium Butyrate Decreases Postweaning Diarrhea by Modulating Intestinal Permeability and Changing the Bacterial Communities in Weaned Piglets. J. Nutr..

[12] Yang X., Xin H., Yang C., Yang X. (2018). Impact of essential oils and organic acids on the growth performance, digestive functions and immunity of broiler chickens. Anim. Nutr..

[13] Fascina V.B., Sartori J.R., Gonzales E., De Carvalho F.B., De Souza I.M.G.P., Polycarpo G.D.V., Stradiotti A.C., Pelícia V.C. (2012). Phytogenic additives and organic acids in broiler chicken diets. Rev. Bras. Zootec..

[14] Tsiloyiannis V.K., Kyriakis S.C., Vlemmas J., Sarris K. (2001). The effect of organic acids on the control of porcine post-weaning diarrhea. Res. Vet. Sci..

[15] Dittoe D.K., Ricke S.C., Kiess A.S. (2018). Organic Acids and Potential for Modifying the Avian Gastrointestinal Tract and Reducing Pathogens and Disease. Front. Vet. Sci..

[16] Pearlin B.V., Muthuvel S., Govidasamy P., Villavan M., Alagawany M., Farag M.R., Dhama K., Gopi M. (2020). Role of acidifiers in livestock nutrition and health: A review. J. Anim. Physiol. Anim. Nutr..

[17] Ravindran V., Kornegay E.T. (1993). Acidification of weaner pig diets: A review. J. Sci. Food Agric..

[18] Nguyen D.H., Seok W.J., Kim I.H. (2020). Organic Acids Mixture as a Dietary Additive for Pigs—A Review. Animals.

[19] De Lange C., Pluske J., Gong J., Nyachoti C. (2010). Strategic use of feed ingredients and feed additives to stimulate gut health and development in young pigs. Livest. Sci..

[20] Jongbloed A., Mroz Z., van der Weij-Jongbloed R., Kemme P. (2000). The effects of microbial phytase, organic acids and their interaction in diets for growing pigs. Livest. Prod. Sci..

[21] Boling S.D., Webel D.M., Mavromichalis I., Parsons C.M., Baker D.H. (2000). The effects of citric acid on phytate-phosphorus utilization in young chicks and pigs. J. Anim. Sci..

[22] Kirchegessner M., Roth F.X. (1982). Fumaric acid as a fed additive in pig nutrition. Pig News Inf..

[23] Ao T. (2005). Exogenous Enzymes and Organic Acids in the Nutrition of Broiler Chicks: Effects on Growth Performance and In Vitro and In Vivo Digestion. Ph.D. Thesis.

[24] Afsharmanesh M., Pourreza J. (2005). Effects of calcium, citric acid, ascorbic acid, vitamin D3 on the efficacy of mi-crobial phytase in broiler starters fed wheat-based diets I. Performance, bone mineralization and ileal digestibility. Int. J. Poult. Sci..

[25] Adil S., Banday T., Bhat G.A., Mir M.S., Rehman M. (2010). Effect of Dietary Supplementation of Organic Acids on Performance, Intestinal Histomorphology, and Serum Biochemistry of Broiler Chicken. Vet. Med. Int..

[26] Van Der Sluis W. (2002). Water quality is important but often overestimated. World Poult..

[27] Omogbenigun F.O., Nyachoti C.M., Slominski B.A. (2003). The effect of supplementing microbial phytase and organic acids to a corn-soybean based diet fed to early-weaned pigs. J. Anim. Sci..

[28] Skřivanová E., Marounek M., Benda V., Březina P. (2006). Susceptibility of Escherichia coli, Salmonella sp and Clostridium perfringens to organic acids and monolaurin. Vet. Med. Praha.

[29] Biagi G., Piva A., Hill T., Schneider D.K., Crenshaw T.D., Ball R. (2003). Low buffering capacity diets with added organic acids as substitute for antibiotics in diets for weaned pigs. Proceedings of the 9th International Symposium on Digestive Physiology in Pigs, Banff, AB, Canada, 14–18 May 2003.

[30] Pinheiro V., Mourao J.L., Alves A., Rodrigues M., Saavedra M.J. Effect of Zinc bacitracin on the performance, digestibility and caecal development of growing rabbits. Proceedings of the 8th World Rabbit Congress.

[31] Mroz Z., Koopmans S.J., Bannink A., Partanen K., Krasucki W., Overland M., Radcliffe S., Mosenthin R., Zentek J., Zebrowska T. (2006). Carboxylic acids as bio regulators and gut growth promoters in non-ruminants. Biology of Nutrition in Growing Animals.

[32] Stratford M., Eklund T. (2003). Organic acids and esters. Food Preservatives.

[33] Suiryanrayna M.V.A.N., Ramana J.V. (2015). A review of the effects of dietary organic acids fed to swine. J. Anim. Sci. Biotechnol..

[34] Dibner J., Buttin P. (2002). Use of organic acids as a model to study the impact of gut microflora on nutrition and metabolism. J. Appl. Poult. Sci..

[35] Luckstadt C., Mellor S. (2011). The use of organic acids in animal nutrition, with special focus on dietary potassium diformate under European and Austral-Asian conditions. Recent Adv. Anim. Nutr. Aust..

[36] Gauthier R. Intestinal health, the key to productivity: The case of organic acids. Proceedings of the XXVII Convention ANECA-WPDC.

[37] Hirshfield I.N., Terzulli S., O’Byrne C. (2003). Weak Organic Acids: A Panoply of Effects on Bacteria. Sci. Prog..

[38] Ng W.-K., Koh C.-B. (2016). The utilization and mode of action of organic acids in the feeds of cultured aquatic animals. Rev. Aquac..

[39] Van Immerseel F., Russell J.B., Flythe M., Gantois I., Timbermont L., Pasmans F., Haesebrouck F., Ducatelle R. (2006). The use of organic acids to combatSalmonellain poultry: A mechanistic explanation of the efficacy. Avian Pathol..

[40] Fernández-Rubio C., Ordóñez C., Abad-González J., Garcia-Gallego A., Honrubia M.P., Mallo J.J., Balana-Fouce R. (2009). Butyric acid-based feed additives help protect broiler chickens from Salmonella Enteritidis infection. Poult. Sci..

[41] Grilli E., Piva A., Callaway T.R., Edrington T.S. (2012). Organic acids and their role in reduce foodborne pathogens in food animals. On-Farm Strategies to Control Foodborne Pathogens.

[42] Grilli E., Vitari F., Domeneghini C., Palmonari A., Tosi G., Fantinati P., Massi P., Piva A. (2013). Development of a feed additive to reduce caecal Campylobacter jejuni in broilers at slaughter age: From in vitro to in vivo, a proof of concept. J. Appl. Microbiol..

[43] Grilli E., Tugnoli B., Passey J.L., Stahl C.H., Piva A., Moeser A.J. (2015). Impact of dietary organic acids and botanicals on intestinal integrity and inflammation in weaned pigs. BMC Vet. Res..

[44] Gheisar M.M., Hosseindoust A., Kim I.H. (2015). Evaluating the effect of microencapsulated blends of organic acids and essential oils in broiler chickens diet. J. Appl. Poult. Res..

[45] Gheisari A.A., Heidari M., Kermanshahi R.K., Togani M., Saraeian S. Effect of dietary supplementation of protected organic acids on ileal microflora and protein digestibility in broiler chickens. Proceedings of the 16th European Symposium on Poultry Nutrition.

[46] Stamilla A., Russo N., Messina A., Spadaro C., Natalello A., Caggia C., Randazzo C.L., Lanza M. (2020). Effects of Microencapsulated Blend of Organic Acids and Essential Oils as a Feed Additive on Quality of Chicken Breast Meat. Animals.

[47] Piva A., Pizzamiglio V., Morlacchini M., Tedeschi M., Piva G. (2007). Lipid microencapsulation allows slow release of organic acids and natural identical flavors along the swine intestine. J. Anim. Sci..

[48] Huyghebaert G., Ducatelle R., Van Immerseel F. (2011). An update on alternatives to antimicrobial growth promoters for broilers. Vet. J..

[49] Tsai C.-C., Hsih H.-Y., Chiu H.-H., Lai Y.-Y., Liu J.-H., Yu B., Tsen H.-Y. (2005). Antagonistic activity against Salmonella infection in vitro and in vivo for two Lactobacillus strains from swine and poultry. Int. J. Food Microbiol..

[50] Baurhoo B., Letellier A., Zhao X., Ruiz-Feria C. (2007). Cecal Populations of Lactobacilli and Bifidobacteria and Escherichia coli Populations After In Vivo Escherichia coli Challenge in Birds Fed Diets with Purified Lignin or Mannanoligosaccharides. Poult. Sci..

[51] Biggs P., Parsons C.M. (2008). The Effects of Grobiotic-P on Growth Performance, Nutrient Digestibilities, and Cecal Microbial Populations in Young Chicks. Poult. Sci..

[52] Papatsiros V.G. (2013). Alternatives to antibiotics for farm animals. CAB Rev. Perspect. Agric. Vet. Sci. Nutr. Nat. Resour..

[53] Fuller R., Fuller R., Heidt P.J., Rusch V., van der Waaij D. (1995). Probiotics: Their development and use. Old Herborn University Seminar Monograph.

[54] Farooq U., Mohsin M., Liu X., Zhang H. (2013). Enhancement of Short Chain Fatty Acid Production from Millet Fibres by Pure Cultures of Probiotic Fermentation. Trop. J. Pharm. Res..

[55] Rehman H., Hellweg P., Taras D., Zentek J. (2008). Effects of Dietary Inulin on the Intestinal Short Chain Fatty Acids and Microbial Ecology in Broiler Chickens as Revealed by Denaturing Gradient Gel Electrophoresis. Poult. Sci..

[56] Walsh M.C., Sholly D.M., Hinson R.B., Saddoris K.L., Sutton A.L., Radcliffe J.S., Odgaard R., Murphy J., Richert B.T. (2007). Effects of water and diet acidification with and without antibiotics on weanling pig growth and microbial shedding. J. Anim. Sci..

[57] Kathrin B. (2009). Benzoic Acid as Feed Additive in Pig Nutrition: Effects of Diet Composition on Performance, Digestion and Ecological Aspects. Ph.D. Thesis.

[58] Falkowski J.F., Aherne F.X. (1984). Fumaric and Citric Acid as Feed Additives in Starter Pig Nutrition. J. Anim. Sci..

[59] Kuang Y., Wang Y., Zhang Y., Song Y., Zhang X., Lin Y., Che L., Xu S., Wu D., Xue B. (2015). Effects of dietary combinations of organic acids and medium chain fatty acids as a replacement of zinc oxide on growth, digestibility and immunity of weaned pigs. Anim. Feed Sci. Technol..

[60] Grecco H.A., Amorim A.B., Saleh M.A., Tse M.L., Telles F.G., Miassi G.M., Pimenta G.M., Berto D.A. (2018). Evaluation of growth performance and gastro-intestinal parameters on the response of weaned piglets to dietary organic acids. An. Acad. Bras. Ciênc..

[61] Risley C.R., Kornegay E.T., Lindemann M.D., Wood C.M., Eigel W.N. (1992). Effect of feeding organic acids on selected intestinal content measurements at varying times post weaning in pigs. J. Anim. Sci..

[62] Htoo J., Molares J. (2012). Effects of dietary supplementation with two potassium formate sources on performance of 8- to 22-kg pigs. J. Anim. Sci..

[63] Canibe N., Højberg O., Højsgaard S., Jensen B.B. (2005). Feed physical form and formic acid addition to the feed affect the gastrointestinal ecology and growth performance of growing pigs. J. Anim. Sci..

[64] Partanen K., Siljander-Rasi H., Alaviuhkola T., Suomi K., Fossi M. (2002). Performance of growing-finishing pigs fed mediumor high-fibre diets supplemented with avilamycin, formic acid or formic acid-sorbate blend. Livest. Prod. Sci..

[65] Yang C., Zhang L., Cao G., Feng J., Yue M., Xu Y., Dai B., Han Q., Guo X. (2019). Effects of dietary supplementation with essential oils and organic acids on the growth performance, immune system, fecal volatile fatty acids, and microflora community in weaned piglets. J. Anim. Sci..

[66] Adil S., Banday T., Bhat G.A., Salahuddin M., Raquib M., Shanaz S. (2011). Response of Broiler Chicken to Dietary Supplementation of Organic Acids. J. Cent. Eur. Agric..

[67] Hassan H.M.A., Mohamed M.A., Youssef A.W., Hassan E.R. (2010). Effect of using organic acids to substitute antibiotic growth promoters on performance and intestinal microflora of broilers. Asian Australas. J. Anim. Sci..

[68] Kamal A.M., Ragaa N.M. (2014). Effect of dietary supplementation of organic acids on performance and serum biochemistry of broiler chicken. Nat. Sci..

[69] Ghazala A.A., Atta A.M., Elkloub K., Mustafa M.E.L., Shata R.F.H. (2011). Effect of dietary supplementation of organic acids on performance, nutrients digestibility and health of broiler chicks. Int. J. Poult. Sci..

[70] Yang X., Liu Y., Yan F., Yang C., Yang X. (2019). Effects of encapsulated organic acids and essential oils on intestinal barrier, microbial count, and bacterial metabolites in broiler chickens. Poult. Sci..

[71] Yang Y., Lee K.Y., Kim I. (2019). Effects of dietary protected organic acids on growth performance, nutrient digestibility, fecal microflora, diarrhea score, and fecal gas emission in weanling pigs. Can. J. Anim. Sci..

[72] Upadhaya S.D., Lee K.Y., Kim I.H. (2016). Effect of protected organic acid blends on growth performance, nutrient digestibility and faecal micro flora in growing pigs. J. Appl. Anim. Res..

[73] Xu Y., Liu L., Long S., Pan L., Piao X. (2018). Effect of organic acids and essential oils on performance, intestinal health and digestive enzyme activities of weaned pigs. Anim. Feed. Sci. Technol..

[74] Namkung H., Gong M.L.J., Yu H., Cottrill M., De Lange C.F.M. (2004). Impact of feeding blends of organic acids and herbal extracts on growth performance, gut microbiota and digestive function in newly weaned pigs. Can. J. Anim. Sci..

[75] Wei X., Bottoms K., Stein H., Blavi L., Bradley C., Bergstrom J., Knapp J., Story R., Maxwell C., Tsai T. (2021). Dietary Organic Acids Modulate Gut Microbiota and Improve Growth Performance of Nursery Pigs. Microorganisms.

[76] Kluge H., Broz J., Eder K. (2006). Effect of benzoic acid on growth performance, nutrient digestibility, nitrogen balance, gastrointestinal microflora and parameters of microbial metabolism in piglets. J. Anim. Physiol. Anim. Nutr..

[77] Li Z., Yi G., Yin J., Sun P., Li D., Knight C. (2008). Effects of Organic Acids on Growth Performance, Gastrointestinal pH, Intestinal Microbial Populations and Immune Responses of Weaned Pigs. Asian Australas. J. Anim. Sci..

[78] Upadhaya S., Lee K., Kim I. (2014). Influence of protected organic acid blends and diets with different nutrient densities on growth performance, nutrient digestibility and faecal noxious gas emission in growing pigs. Vet. Med..

[79] Øverland M., Kjos N., Borg M., Skjerve E., Sørum H. (2008). Organic acids in diets for entire male pigs: Effect on skatole level, microbiota in digesta, and growth performance. Livest. Sci..

[80] Papatsiros V.G., Tassis P.D., Tzika E.D., Papaioannou D.S., Petridou E., Alexopoulos C., Kyriakis S.C. (2011). Effect of benzoic acid and combination of benzoic acid with a probiotic containing Bacillus Cereus var. toyoi in weaned pig nutrition. Pol. J. Vet. Sci..

[81] Saleem K., Saima, Rahman A., Pasha T.N., Mahmud A., Hayat Z. (2020). Effects of dietary organic acids on performance, cecal microbiota, and gut morphology in broilers. Trop. Anim. Health Prod..

[82] Ali A.M., El Agrab H.M., Hamoud M.M., Gamal A.M., Mousa M.R., Nasr S.A.E., El Shater M.A.H., Laban S.E., Zahran O.K., Ali M.M. (2020). Effect of Acidified Drinking Water by Organic Acids on Broiler Performance and Gut Health. Adv. Anim. Vet. Sci..

[83] Stamilla A., Messina A., Sallemi S., Condorelli L., Antoci F., Puleio R., Loria G.R., Cascone G., Lanza M. (2020). Effects of Microencapsulated Blends of Organics Acids (OA) and Essential Oils (EO) as a Feed Additive for Broiler Chicken. A Focus on Growth Performance, Gut Morphology and Microbiology. Animals.

[84] Goh C.H., Loh T.C., Foo H.L., Nobilly F. (2020). Fecal Microbial Population and Growth in Broiler Fed Organic Acids and Palm Fat-Composed Diet. Trop. Anim. Sci. J..

[85] Dai D., Qiu K., Zhang H.-J., Wu S.-G., Han Y.-M., Wu Y.-Y., Qi G.-H., Wang J. (2021). Organic Acids as Alternatives for Antibiotic Growth Promoters Alter the Intestinal Structure and Microbiota and Improve the Growth Performance in Broilers. Front. Microbiol..

[86] Sabour S., Tabeidian S.A., Sadeghi G. (2019). Dietary organic acid and fiber sources affect performance, intestinal morphology, immune responses and gut microflora in broilers. Anim. Nutr..

[87] Fathi R., Samadi M.S., Qotbi A.A., Seidavi A., Marín A.L.M. (2016). Effects of feed supplementation with increasing levels of organic acids on growth performance, carcass traits, gut microbiota and pH, plasma metabolites, and immune response of broilers. Anim. Sci. Pap. Rep..

[88] Panda A.K., Rao S.V.R., Raju M.V.L.N., Sunder G.S. (2009). Effect of Butyric Acid on Performance, Gastrointestinal Tract Health and Carcass Characteristics in Broiler Chickens. Asian Australas. J. Anim. Sci..

[89] Sultan A., Ullah T., Khan S., Khan R.U. (2015). Effect of organic acid supplementation on the performance and ileal microflora of broiler during finishing period. Pak. J. Zool..

[90] Panda A.K., Raju M.V.L.N., Rao S.R., Sunder G.S., Reddy M.R. (2009). Effect of graded levels of formic acid on gut microflora count, serum biochemical parameters, performance and carcass yield of broiler chickens. Indian J. Anim. Sci..

[91] Lakshmi K.V., Sunder G.S. (2015). Supplementation of Propionic Acid (PA), Butyric Acid (BA) or Antibiotic (AB) in diets and their influence on broiler performance, carcass parameters and immune response. IJSR.

[92] Upadhaya S.D., Lee K.Y., Kim I.H. (2014). Protected Organic Acid Blends as an Alternative to Antibiotics in Finishing Pigs. Asian Australas. J. Anim. Sci..

[93] Rosiński S., Grigorescu G., Lewinska D., Ritzén L.G., Viernstein H., Teunou E., Poncelet D., Zhang Z., Fan X., Serp D. (2002). Characterization of microcapsules: Recommended methods based on round-robin testing. J. Microencapsul..

[94] Hossain M., Jayaraman B., Kim S., Lee K., Kim I., Nyachoti C. (2018). Effects of a matrix-coated organic acids and medium-chain fatty acids blend on performance, and in vitro fecal noxious gas emissions in growing pigs fed in-feed antibiotic-free diets. Can. J. Anim. Sci..

[95] Liu S., Hou W., Cheng S., Shi B., Shan A. (2014). Effects of dietary citric acid on performance, digestibility of calcium and phosphorus, milk composition and immunoglobulin in sows during late gestation and lactation. Anim. Feed. Sci. Technol..

[96] Sauer W., Cervantes M., Yanez J., Araiza B., Murdoch G., Morales A., Zijlstra R.T. (2009). Effect of dietary inclusion of benzoic acid on mineral balance in growing pigs. Livest. Sci..

[97] Bühler K., Liesegang A., Bucher B., Wenk C., Broz J. (2010). Influence of benzoic acid and phytase in low-phosphorus diets on bone characteristics in growing-finishing pigs. J. Anim. Sci..

[98] Guggenbuhl P., Séon A., Quintana A.P., Nunes C.S. (2007). Effects of dietary supplementation with benzoic acid (VevoVitall®) on the zootechnical performance, the gastrointestinal microflora and the ileal digestibility of the young pig. Livest. Sci..

[99] Kluge H., Broz J., Eder K. (2010). Effects of dietary benzoic acid on urinary pH and nutrient digestibility in lactating sows. Livest. Sci..

[100] Hernandez F., García V., Madrid J., Orengo J., Catalá P., Megías M.D. (2006). Effect of formic acid on performance, digestibility, intestinal histomorphology and plasma metabolite levels of broiler chickens. Br. Poult. Sci..

[101] García V., Catalá-Gregori P., Hernandez F., Megías M.D., Madrid J. (2007). Effect of Formic Acid and Plant Extracts on Growth, Nutrient Digestibility, Intestine Mucosa Morphology, and Meat Yield of Broilers. J. Appl. Poult. Res..

[102] Ao T., Cantor A.H., Pescatore A.J., Ford M.J., Pierce J.L., Dawson K.A. (2009). Effect of enzyme supplementation and acidification of diets on nutrient digestibility and growth performance of broiler chicks. Poult. Sci..

[103] Lohakare J.D., Ryu M.H., Hahn T.-W., Lee J.K., Chae B.J. (2005). Effects of Supplemental Ascorbic Acid on the Performance and Immunity of Commercial Broilers. J. Appl. Poult. Res..

[104] Emami N.K., Naeini S.Z., Ruiz-Feria C. (2013). Growth performance, digestibility, immune response and intestinal morphology of male broilers fed phosphorus deficient diets supplemented with microbial phytase and organic acids. Livest. Sci..

[105] Smulikowska S., Czerwiński J., Mieczkowska A., Jankowiak J. (2009). The effect of fat-coated organic acid salts and a feed enzyme on growth performance, nutrient utilization, microflora activity, and morphology of the small intestine in broiler chickens. J. Anim. Feed. Sci..

[106] Stefanello C., Rosa D.P., Dalmoro Y.K., Segatto A.L., Vieira M.S., Moraes M.L., Santin E. (2020). Protected Blend of Organic Acids and Essential Oils Improves Growth Performance, Nutrient Digestibility, and Intestinal Health of Broiler Chickens Undergoing an Intestinal Challenge. Front. Vet. Sci..

[107] Centeno C., Arija I., Viveros A., Brenes A. (2007). Effects of citric acid and microbial phytase on amino acid digestibility in broiler chickens. Br. Poult. Sci..

[108] Cho J.H., Song M.H., Kim I.H. (2014). Effect of microencapsulated blends of organic acids and essential oils supplementation on growth performance and nutrient digestibility in finishing pigs. Rev. Colomb. Cienc. Pecu..

[109] Wang J.P., Yoo J.S., Lee J.H., Jang H.D., Kim H.J., Shin S.O., Seong S.I. (2009). Effects of phenyllactic acid on growth performance, nutrient digestibility, microbial shedding, and blood profile in pigs. J. Anim. Sci..

[110] Long S., Xu Y., Pan L., Wang Q., Wang C., Wu J., Wu Y., Han Y., Yun C., Piao X. (2018). Mixed organic acids as antibiotic substitutes improve performance, serum immunity, intestinal morphology and microbiota for weaned piglets. Anim. Feed. Sci. Technol..

[111] Gerritsen R., van Dijk A., Rethy K., Bikker P. (2010). The effect of blends of organic acids on apparent faecal digestibility in piglets. Livest. Sci..

[112] Mroz Z., Jongbloed A.W., Partanen K.H., Vreman K., Kemme P.A., Kogut J. (2000). The effects of calcium benzoate in diets with or without organic acids on dietary buffering capacity, apparent digestibility, retention of nutrients, and manure characteristics in swine. J. Anim. Sci..

[113] Devi S.M., Lee K.Y., Kim I.H. (2016). Analysis of the effect of dietary protected organic acid blend on lactating sows and their piglets. Rev. Bras. Zootec..

[114] Ndelekwute E., Afolabi K., Uzegbu H., Essien E. (2015). Effect of dietary formic acid as replacement of streptomycin on growth and nutrient digestibility in broiler. Bangladesh J. Anim. Sci..

[115] Ndelekwute E.K., Unah U.L., Udoh U.H. (2019). Effect of dietary organic acids on nutrient digestibility, faecal moisture, digesta pH and viscosity of broiler chickens. MOJ Anat. Physiol..

[116] Lei X.J., Lee S.I., Lee K.Y., Nguyen D.H., Kim I.H. (2018). Effects of a blend of organic acids and medium-chain fatty acids with and without Enterococcus faecium on growth performance, nutrient digestibility, blood parameters, and meat quality in finishing pigs. Can. J. Anim. Sci..

[117] Jansons I., Jemeljanovs A., Konosonoka I.H., Sterna V., Lujane B. (2011). The influence of organic acid additive, phytoadditive and complex of organic acid additive phytoadditive on pig productivity, meat quality. Agron. Res..

[118] Brzóska F., Sliwiński B., Michalik-Rutkowska O. (2013). Effect of Dietary Acidifier on Growth, Mortality, Post-Slaughter Parameters and Meat Composition of Broiler Chickens/Wpływ zakwaszacza diety na masę ciała, śmiertelność, wydajność rzeźną i skład mięsa kurcząt rzeźnych. Ann. Anim. Sci..

[119] Jha A.K., Azad H., Ali S.N., Alam P., Sheikh N., Ali H., Ansari K. (2019). Evaluation of Growth and Carcass Characteristics of Broiler Chickens (Cobb 500) Feed on Different Level of Organic Acids Inclusion in Diet at Parwanipur. Nepal. Vet. J..

[120] Galli G.M., Aniecevski E., Petrolli T.G., da Rosa G., Boiago M.M., Simões C.A., Wagner R., Copetti P.M., Morsch V.M., Araujo D.N. (2021). Growth performance and meat quality of broilers fed with microencapsulated organic acids. Anim. Feed. Sci. Technol..

[121] Mir N.A., Rafiq A., Kumar F., Singh V., Shukla V. (2017). Determinants of Broiler Chicken Meat Quality and Factors Affecting Them: A Review. J. Food Sci. Technol..

[122] Karunanayaka D.S., Jayasena D.D., Jo C. (2016). Prevalence of pale, soft, and exudative (PSE) condition in chicken meat used for commercial meat processing and its effect on roasted chicken breast. J. Anim. Sci. Technol..

[123] Sugiharto S., Yudiarti T., Isroli I., Widiastuti E., Wahyuni H.I., Sartono T.A., Nurwantoro N., Al-Baarri A.N. (2019). Effect of dietary supplementation of formic acid, butyric acid or their combination on carcass and meat characteristics of broiler chickens. J. Indones. Trop. Anim. Agric..

[124] El-Senousey H.K., Fouad A.M., Yao J.H., Zhang Z.G., Shen Q.W. (2013). Dietary Alpha Lipoic Acid Improves Body Composition, Meat Quality and Decreases Collagen Content in Muscle of Broiler Chickens. Asian Australas. J. Anim. Sci..

[125] Menconi A., Kuttappan V.A., Hernandez-Velasco X., Urbano T., Matté F., Layton S., Kallapura G., Latorre J., Morales B.E., Prado O. (2014). Evaluation of a commercially available organic acid product on body weight loss, carcass yield, and meat quality during preslaughter feed withdrawal in broiler chickens: A poultry welfare and economic perspective. Poult. Sci..

[126] Attia F.M. (2018). Effect of organic acids supplementation on nutrients digestibility, gut microbiota and immune response of broiler chicks. Egypt Poult. Sci. J..

[127] Akbar M.A., Tewatia B.S., Kumar S. (2018). Effect of dietary supplementation of salts of organic acids on gut morphology and meat quality of broilers. Indian J. Anim. Res..

[128] Ben Braïek O., Smaoui S. (2021). Chemistry, Safety, and Challenges of the Use of Organic Acids and Their Derivative Salts in Meat Preservation. J. Food Qual..

[129] Yang X., Wang J., Feng Z., Zhang X., Wang X., Wu Q. (2019). Relation of the pdxB-usg-truA-dedA Operon and the truA Gene to the Intracellular Survival of Salmonella enterica Serovar Typhimurium. Int. J. Mol. Sci..

[130] Bearson S., Bearson B., Foster J.W. (1997). Acid stress responses in enterobacteria. FEMS Microbiol. Lett..

[131] Barua S., Yamashino T., Hasegawa T., Yokoyama K., Torii K., Ohta M. (2002). Involvement of surface polysaccharides in the organic acid resistance of Shiga Toxin-producing Escherichia coli O157:H7. Mol. Microbiol..

[132] Foster J.W. (1993). The acid tolerance response of Salmonella typhimurium involves transient synthesis of key acid shock proteins. J. Bacteriol..

[133] Foster J.W. (1991). Salmonella acid shock proteins are required for the adaptive acid tolerance response. J. Bacteriol..

[134] Kwon Y., Park S., Birkhold S., Ricke S. (2000). Induction of Resistance of Salmonella typhimurium to Environmental Stresses by Exposure to Short-Chain Fatty Acids. J. Food Sci..

[135] Thompson J.L., Hinton M. (1997). Antibacterial activity of formic and propionic acids in the diet of hens on salmonellas in the crop. Br. Poult. Sci..

